# The incidence and prognosis of thymic squamous cell carcinoma: A Surveillance, Epidemiology, and End Results Program population-based study

**DOI:** 10.1097/MD.0000000000025331

**Published:** 2021-04-16

**Authors:** Jingyi Wu, Zhijun Wang, Caibao Jing, Yang Hu, Bing Yang, Yanping Hu

**Affiliations:** Department of Medical Oncology, Hubei Cancer Hospital, Tongji Medical College, Huazhong University of Science and Technology, Wuhan, Hubei, PR China.

**Keywords:** population, prognosis, Surveillance, Epidemiology, and End Results database, survival, thymic squamous cell carcinoma

## Abstract

**Background:**

Thymic carcinoma represents a rare type of malignant mediastinal tumor and has been the subject of controversy. Although independent prognostic factors related to thymic carcinoma have been investigated previously, few studies have focused specifically on the survival outcomes associated with thymic squamous cell carcinoma (TSCC). This study aims at presenting a survival analysis in this rare malignant disease at population level.

**Methods:**

We extracted the data of 216 patients with TSCC recorded from 1973 to 2015 from the Surveillance, Epidemiology, and End Results (SEER) database of the National Cancer Institute. The patients’ demographic features, clinical traits, and treatment factors were analyzed in order to identify prognostic factors, which correlate overall survival using the Kaplan–Meier method as well as a multivariate Cox regression model, for TSCC.

**Results:**

The majority of patients were male, Caucasian, married, and insured. Furthermore, 58.3%, 54.6%, and 59.7% of patients TSCC underwent surgery, radiotherapy, and chemotherapy respectively. In a multivariate analysis, age of diagnosis (hazard ratio [HR]: 1.022, 95% confidence interval [CI]: 1.003–1.040, *P* = .020), surgical treatment (HR: 0.282, 95% CI: 0.164–0.484, *P* = .000), and stage (regional vs distant HR: 0.532, 95% CI: 0.324–0.872, *P* = .013; localized vs distant HR: 0.297, 95% CI: 0.133–0.664, *P* = .003) correlated with increased overall survival, whereas adjuvant therapy, including chemotherapy and radiotherapy, did not correlate with survival. Among surgically treated patients, age of diagnosis and stage were associated with better overall survival, while chemotherapy and radiotherapy did not contribute significantly to overall survival.

**Conclusion:**

Surgery, age of diagnosis, and stage were associated with better overall survival among TSCC.

## 1 Introduction

Thymic carcinoma is a rare and highly malignant type of solid tumor that originates from the thymic epithelium and shows malignant cytological features with aggressive tumor invasiveness and high potential for metastasis. The mortality rate of thymic cancer is still in the top 10 among the chest tumors. Thymic squamous cell carcinoma (TSCC) is the main pathological type of thymic cancer, accounting for approximately 80% of all thymic carcinomas.^[1,2]^ The consensus has not been reached due to the limited number of patients.

Although the latest National Comprehensive Cancer Network (NCCN) has general guidelines for thymic carcinom, but it is not specific for TSCC. However, in a single-center study by Zhao et al^[4]^ enrolled 105 consecutive patients with TSCC who underwent surgery. In the comparison of the patients’ clinical and pathological data, only surgical completeness was found to have a strong effect on the overall survival and to extend disease-free survival^[4]^ along with chemotherapy. Yet still, we need more research to have a consensus specifically on TSCC.

The Surveillance, Epidemiology, and End Results Program (SEER) database collects carcinoma incidence data from population-based cancer registries covering approximately 34.6% of the United State population. In this study, we used data from the SEER database mentioned above to identify the prognostic factors for overall survival among patients with TSCC.

## 2 Materials and methods

The SEER database is sponsored by the National Cancer Institute and has been used to track cancer incidence and survival since 1973. This database covers more than a third of the US population, that is, approximately 86.4 million people (2010 census from SEER official website).^[5]^ We extracted information concerning cases of TSCC recorded during 1973 to 2015 from the SEER database, using SEER∗Stat Software, version 1 (National Cancer Institute, Bethesda, MD). The extracted information included patients’ time of diagnosis, demographics, stage of diagnosis, treatment, and follow-up to determine vital status. Cases of TSCC were identified using codes from the International Classification of Diseases for Oncology, Third Edition (ICD-O-3). First, the tumor primary site was identified as the Mediastinum (C339, C381–C383, C388, C390, C398, C399) or other endocrine, including thymic (C379, C740–C749, C750–C759). The histology was identified as squamous cell carcinoma (8070/3, 8070/6, 8071/3, 8072/3, 8073/3, 8074/3, 8075/3, 8076/2, 8076/3, 8078/3). The selected cases of thymic carcinoma were then restricted to only TSCC. Patients without a documented vital status at the end of follow-up (2015/12) were excluded.

The acquired data were input and organized into spreadsheets using Excel 2016 (Microsoft Corp., Redmond, WA). The information obtained included the time of diagnosis, sex, age of diagnosis, ethnicity, marital status, surgery data, chemotherapy data, radiotherapy data, SEER stage, cause of death, insurance, and survival duration. Given the small sample size, we established groups, each containing few samples for every factor. Patients were stratified by race/ethnicity as Caucasian, Asian or Pacific Islander, and others (unknown and African). The proportions of sexes and races/ethnicities were calculated and compared with the 2000 US standard population in the SEER database. Marital status was categorized into married or unmarried (single, divorced, widowed, or separated during the time of diagnosis). SEER stage was classified as localized disease (confined in the original organ without extension beyond the primary organ), regional disease (direct extension to adjacent organs or structures or by spreading to regional lymph nodes), or distant disease (spreading to parts of the body remote from the primary tumor). The primary end points of the study were overall survival (OS) and cancer-specific survival (CSS). Overall survival was calculated from the time of diagnosis to the time of death of any cause, while cancer-specific survival was calculated from the time of diagnosis to the time of death only of TSCC, and both durations were estimated by the Kaplan–Meier method. A Cox proportional hazard model of survival-associated risk factors was applied to conducting univariable and multivariable analyses and generating hazard ratios (HRs) with corresponding 95% confidence intervals (CIs). Two-sided *P*-values <.05 were taken in to significant consideration statistically, and the survival curves were estimated also by the Kaplan–Meier method. A nomogram based on possible prognostic factors associated with OS was established using R software, on the basis of the Cox regression model, and its performance was measured by concordance index (*c*-index) and assessed by calibration curves. The concordance index (*c*-index), which can reflect the probability of the concordance between observed and predicted outcome, was used to evaluate the discrimination of nomograms.^[6]^ The calibration curve was used to compare the predicted and observed probabilities of survival in the cohort study. Nomograms and calibration curves were developed along with bootstraps of 200 resamples to reduce the overfit bias. All statistical analyses will employ SPSS Statistics 20.0 (IBM, Inc., Armonk, NY).

Hubei Cancer Hospital Ethics Committee has approved the study. The patients in SEER database were informed of information collection and were consent to participate in the medical research.

## 3 Results

### 3.1 Demographics, clinical traits, and treatments

In our analysis of the SEER database, we identified 216 patients who were diagnosed with TSCC from 1973 to 2015. The patients had a median age of 54 years, and a bit more men were observed than women (54.6% vs 50.9% in the general population) (US Census, year 2010).^[5]^ Furthermore, the cohort exhibited a significant predilection for Asian and Caucasian patients, who comprised 23.0% (compared with 8.5% in the US Census for 2000) and 83.6% (compared with 65.3% in the US Census for 2000) respectively. Regarding tumor characteristics, 46.3% of cases were classified as regional stage, while 31.6% were distant stage. Further details are listed in Table 1. According to the SEER database, the most common treatment option was surgery, which was chosen in 58.3% of cases. In addition to surgery, 54.6% and 59.7% of the cases received radiotherapy and chemotherapy respectively.

**Table 1:** Characteristics of thymic squamous cell cancer in SEER database.

	Number of patients	Percent of total number
Total number	216	100.0%
Mean age of diagnosis = 62 years		
Time of diagnosis		
1997–2000	21	9.7%
2000–2010	74	34.2%
2010–2015	121	56.1%
Gender		
Female	98	45.4%
Male	118	54.6%
Race		
White	141	65.3%
Asian or Pacific Islander	51	23.6%
Others	24	11.1%
Marital status		
Married	135	62.5%
Single	70	32.4%
Unknown	11	5.1%
SEER stage		
Localized	42	19.4%
Regional	100	46.3%
Distant	69	31.9%
Unknown	5	2.4%
Surgery		
Yes	126	58.3%
None/Unknown	90	41.7%
Radiotherapy		
Yes	118	54.6%
None/Unknown	98	45.4%
Chemotherapy		
Yes	129	59.7%
None/Unknown	87	40.3%
Insurance		
Insured	143	66.2%
Uninsured/unknown	73	33.8%

### 3.2 Survival

**Figure 1:**
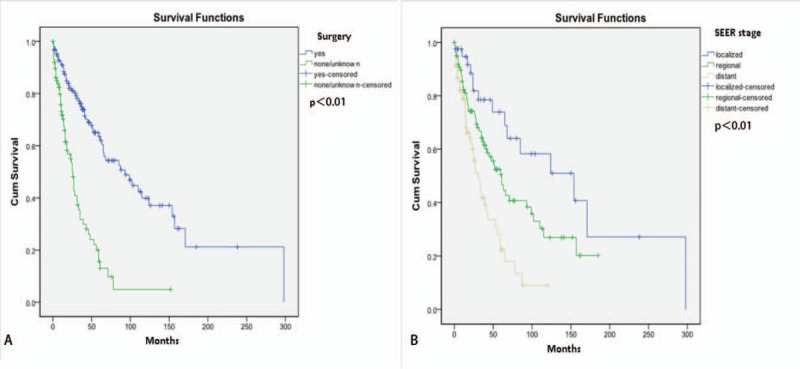
Overall survival curves of TSCC according to: patients in different stages (A); patients with and without surgery (B); all *P* < .05. TSCC = thymic squamous cell carcinoma.

We used the Kaplan–Meier method and a stratified Cox proportional hazards model to evaluate the distributions of overall survival according to different variables. In the univariable analysis, we observed a significant difference in overall survival of non-surgery and surgery, with respective durations of 25 and 93 months. In the multivariable analysis, surgery was identified as a significant and independent prognostic factor for overall survival (HR: 0.282, 95% CI: 0.164–0.484, *P* = .000). In an age-stratified analysis, the risk of death increased by 2.2% every year and a multivariable Cox regression identified the age of diagnosis as an independent prognostic factor for survival (hazard ratio [HR]: 1.022, 95% CI: 1.003–1.040, *P* = .020). Moreover, patients with local-stage disease had a longer overall survival compared with those with regional- and distant-stage disease, and SEER stage was also found to have significant correlation with overall survival in a multivariable analysis (regional vs distant HR: 0.532, 95% CI: 0.324–0.872, *P* = .013; localized vs distant HR: 0.297, 95% CI: 0.133–0.664, *P* = .003). Kaplan–Meier analyses showed that surgical treatment and localized stage had better outcomes for OS (Fig. 1A and B). Furthermore, chemotherapy and radiotherapy did not show the ability to yield any significant benefits in terms of overall survival. Further details are listed in Table 2.

**Table 2:** Univariable and multivariable analysis of variables of overall survival in TSCC.

	Univariable analysis		Multivariable analysis	
Variable	HR (95% CI)	*P*	HR (95% CI)	*P*
Time of diagnosis	1.004 (0.976–1.032)	.800	0.978 (0.935–1.023)	.331
Age of diagnosis	1.007 (0.992–1.023)	.346	1.022 (1.003–1.040)	.020
Gender				
Male	ref		ref	
Female	1.372 (0.943–1.997)	.098	1.488 (0.943–2.350)	.088
Race				
Asian or Pacific Islander	ref		ref	
White	0.903 (0.712–1.145)	.400	1.028 (0.615–1.718)	.915
Marital status				
Unmarried	ref		ref	
Married	0.731 (0.492–1.085)	.120	1.022 (0.645–1.644)	.929
SEER stage				
Distant	ref		ref	
Regional	0.511 (0.337–0.777)	.002	0.532 (0.324–0.872)	.013
Localized	0.266 (0.142–0.499)	.000	0.297 (0.133–0.664)	.003
Surgery				
None/Unknown	ref		ref	
Yes	3.590 (2.411–5.345)	.000	0.282 (0.164–0.484)	.000
Radiotherapy				
None/Unknown	ref		ref	
Yes	1.344 (0.927–1.949)	.116	1.188 (0.743–1.900)	.472
Chemotherapy				
None/Unknown	ref		ref	
Yes	0.594 (0.400–0.882)	.010	1.166 (0.682–1.993)	.575
Insurance				
Uninsured/unknown	ref		ref	
Insured	1.083 (0.726–1.616)	.696	0.812 (0.432–1.526)	.517
Quamous cell cancer				

### 3.3 The patients with TSCC who underwent surgical therapy

Surgical therapy was performed in 126 patients, among whom with localized stage and regional stage or of younger age were more likely to have better survival. However, the radiotherapy and chemotherapy may not contribute to longer survival for the patients with surgery (Table 3).

**Table 3:** Multivariable analysis of variables of overall survival in thymic squamous cell cancer based on the surgery.

	Mean overall survival, mo	HR (95% CI)	*P*
Age of diagnosis	–	1.031 (1.001–1.062)	.044
Time of diagnosis			
2010–2015	54	ref	–
2000–2010	103	2.346 (0681–8.074)	.176
1997–2000	102	5.807 (1.160–29.079)	.032
Gender			
Male	115	ref	–
Female	120	0.515 (0.255–1.041)	.065
Race			
Asian or Pacific Islander	146	ref	–
White	104	1.448 (0.676–3.100)	.340
Marital status			
Unmarried	98	ref	–
Married	121	0.472 (0.205–1.084)	.077
SEER stage			
Distant	54	ref	–
Regional	98	0.414 (0.192–0.892)	.024
Localized	159	0.169 (0.058–0.493)	.001
Radiotherapy			
None/Unknown	116	ref	–
Yes	119	1.054 (0.515–2.158)	.885
Chemotherapy			
None/Unknown	131	ref	–
Yes	89	1.743 (0.886–3.429)	.107
Insurance			
Uninsured/unknown	119	ref	–
Insured	70	1.752 (0.568–5.406)	.329

### 3.4 Prognostic nomogram for OS

**Figure 2:**
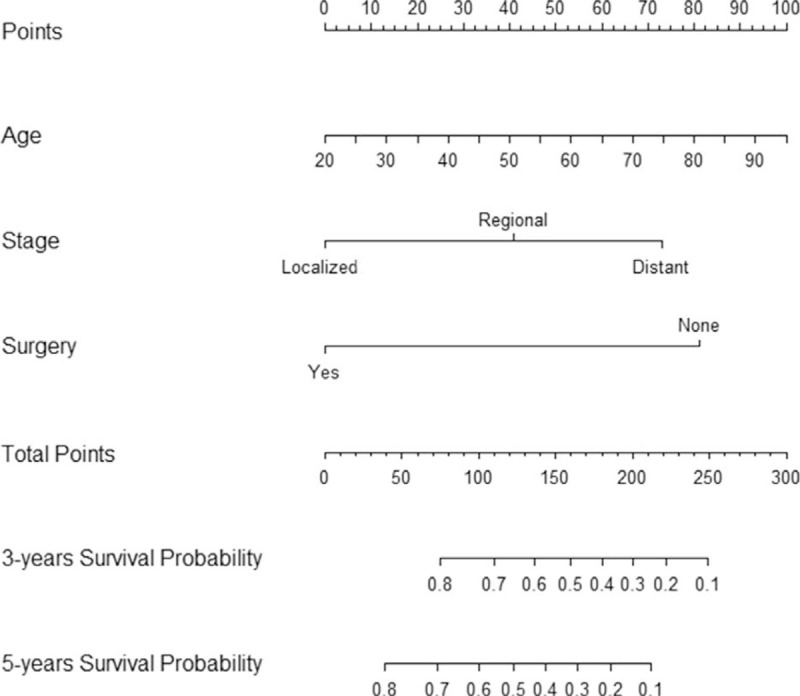
Nomogram to predict the probability of 3-year OS and 5-year OS. OS = overall survival.

**Figure 3:**
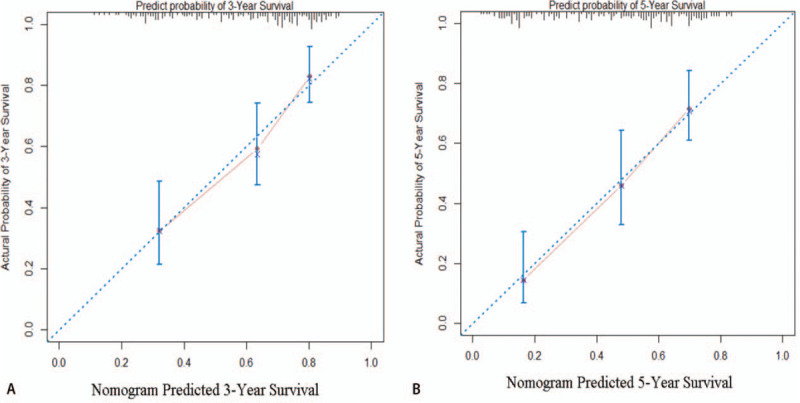
Calibration plot showing nomogram-predicted 3-year OS probabilities with the actual 3-year OS (A) and the nomogram-predicted 5-year OS with the actual 5-year OS (B). OS = overall survival.

A prognostic nomogram that corporates all significant prognostic factors in multivariate analysis for OS is shown in Fig. 2. The *c*-index of prognostic nomogram for OS prediction was 0.566 (95% CI, 0.492–0.640) in the cohort. The calibration plot for the probability of OS at 3 or 5 years shows a positive correlation between the nomogram prediction and actual observation (Fig. 3).

## 4 Discussion

TSCC is the most common histological subtype of thymic carcinoma, accounting for approximately 1 in every 5 cases.^[1,7]^ However, few studies have focused on specifically on survival among patients with TSCC. Notably, we found that age of diagnosis, surgical treatment, and SEER stage were associated with better prognosis. To our knowledge, this is the largest existing research study at population level to present clinical and pathology information regarding TSCC.

Currently, patients with TSCC have a relatively good overall prognosis, with median survival duration of 60 months and 1-, 3-, and 5-year survival rates of 83%, 55%, and 36% patients, respectively. SEER database with existing literature showed the median survival of the 290 included cases of thymic carcinoma was 48 months, and the 5-year survival rate was slightly better than that of TSCC.^[8]^ This discrepancy has been attributed to differences in pathological types.

In all previous studies of thymic carcinoma, surgery has been the preferred curative treatment, with 5-year survival rates ranging between 58% and 80%.^[8–12]^ Consistent with those findings and the NCCN guideline, our study observed the same survival benefits of surgery, with a 3-year overall survival rates of 73%. In a survival analysis of 105 surgically treated cases of TSCC, Zhao study reported median survival duration of 86.1 months after complete resection as well as 50.6 months after incomplete resection, and demonstrated that only the former had a significant impact on overall survival.^[4]^ Suster et al^[20]^ research also confirms the significance of the surgery. Undoubtedly, surgery remains the first choice for the treatment of TSCC. However, we must not overlook the fact that patients who choose to take surgery are more likely to be in a good condition and have localized and regional disease.

It is also worth noting that we identified the age of diagnosis as an independent prognostic factor for overall survival among patients with TSCC. In this way, we analyze age as a continuous variable, which distinguishes our study from other researchers such as Zhao study, who selected a cut-off age value of 55 years in their analysis of surgical TSCC,^[4]^ and Yasuko study, who selected 60 years as a cut-off age value in a study of resected thymic carcinoma.^[12]^ However, neither of the 2 studies identified age as a significant prognostic factor for survival. In line with our study, in a propensity-matched analysis of thymic carcinoma,^[13]^ significantly poorer survival was seen in patients with age ≥63 years (*P* = .008). We are currently not able to confirm those findings, but elderly people are suffering from a gradually increasing frailty and prone to accept palliative treatment to reduce their pain. Differences in therapeutic treatments may contribute to differences of survival time.

The most common stage of TSCC is Masaoka staging. Prognostic impact of Masaoka stage has been described in the previous studies.^[7,8,12]^ As we know, Masaoka stage is still the most consistent prognostic factor so far. However, there is unique staging style provided by SEER database, which is localized, regional, distant stage. In our study, the SEER stage showed its benefits of overall survival. Zhai et al^[14]^ also found the percentage of local-regional relapse free survivals were 81.4% and 54.4%, and that of distant metastasis-free survivals were 35.9% and 25.6% at 5 and 10 years. Localized stage is more likely to accept surgical treatment, just as the outcomes in our study, 78.6% of localized stage accept surgery which is far more than regional stage (63%) and distant stage (43%). This is probably the most important reason why stage was significantly related to the overall survival.

The ability of adjuvant therapy to improve overall survival remains controversial. According to our study, neither radiotherapy nor chemotherapy contributed to longer overall survival. Similarly, Zhao study reported that postoperative adjuvant therapy, which was comprised of radiotherapy or chemotherapy alone or in combination, did not yield overall survival benefits to patients with TSCC.^[4]^ Other studies of thymic carcinoma, however, have reported different findings.^[12,13,15,16]^ For example, a meta-analysis of 973 patients by Hamaji et al^[17]^ demonstrated that postoperative radiotherapy (PORT) after thymic carcinoma resection could lead to improved overall and progression-free survival. Besides, Yen et al^[18]^ reported that among 82 patients with recurrent thymic carcinoma after surgical intervention, postoperative chemotherapy yielded the best progression-free survival outcomes after recurrence. In a study of 86 cases of advanced thymic carcinoma by Song et al,^[19]^ the objective response rate to first-line chemotherapy was 47.7%, and the disease control rate was 80.2%. Yabuki and Minowa^[21]^ reported a case of long survival and recurrence of thymic carcinoma 10 years after resection followed by concurrent chemoradiotherapy. In other words, adjuvant therapy may still play a pivotal role in the treatment of thymic carcinoma. However, our study failed to demonstrate an overall survival benefit of adjuvant therapy for patients with TSCC. Still, for patients with advanced TSCC who do not meet the indications for surgery, adjuvant therapy should be considered for disease control or palliative use.

It is universally acknowledged that the death and incidence rate of some tumors are often related with race/ethinicity,^[22–25]^ therefore, in our study, we also discuss whether race/ethnicity is associated with the survival of TSCC, and discovers that race/ethnicity does not have any correlation with the overall survival. The Gad MM's study has proposed that pancreatic adenocarcinoma is associated with race and confirmed the annual increasing mortality rate of Asians, which does not bear any correlation with age and stage.^[25]^ It can be argued that the tumors related with race/ethnicity are such as lung cancer, colorectal cancer, cholangiocarcinoma, and high-grade glioma, which is relevant to their diet, lifestyle, and local social economy.^[23]^ In the meantime, it is proved in the study of Elisabeth that these tumors mentioned before have significant differences and trends in molecular signatures of the 3 cancer types in African Americans and Caucasian cohorts.^[24]^ As for TSCC, though its incidence rate might have little relation with the factors above, the study of its overall survival also corroborates that the incidence rate and mortality rate of TSCC has nothing to do with race.

Our study had some limitations of note. We were only able to describe the presence or absence of surgery, radiotherapy, and chemotherapy, as the SEER database does not provide information about the type of surgery (e.g., complete or incomplete resection or pathological biopsy). Furthermore, we lacked information about the types of radiotherapy and chemotherapy the patients received, including the details of the radiotherapy methods, total doses, daily fraction sizes, irradiation field volumes, and chemotherapy regimens. Moreover, the database lacked information about the pathologic resection margins, which inhibited the analysis of postoperative radiotherapy outcomes, as well as detailed Masaoka stage information, which denotes prognosis. Despite these limitations, the SEER database is an invaluable resource with which to study rare malignancies, especially TSCC. Our study is the first multicenter study of TSCC and thus provides insights into the epidemiology and survival prognostic factors of this type of malignancy. Hopefully, our findings will inform the clinical practice with our recommendations.

## 5 Conclusion

TSCC is a rare type of carcinoma for which surgery should be considered as the best primary treatment option. Notably, the age of diagnosis and SEER stage were independent prognostic factors related to overall survival. Further studies are needed to confirm the effects of adjuvant therapy and better understand the effects of these treatments on overall survival when they are administered postoperatively to patients with TSCC. We hope that our findings will help to identify more appropriate and practical treatments for TSCC.

## Acknowledgments

The authors would like to thank SEER for open access to their database.

## Author contributions

**Conceptualization:** Jingyi Wu, Yanping Hu.

**Data curation:** Jingyi Wu, Yanping Hu.

**Formal analysis:** Jingyi Wu, Yanping Hu.

**Funding acquisition:** Jingyi Wu, Zhijun Wang, Caibao Jing, Yanping Hu.

**Investigation:** Jingyi Wu, Caibao Jing, Yanping Hu.

**Methodology:** Jingyi Wu, Zhijun Wang, Yanping Hu.

**Project administration:** Jingyi Wu, Zhijun Wang, Yang Hu, Yanping Hu.

**Resources:** Jingyi Wu, Yang Hu, Bing Yang, Yanping Hu.

**Software:** Jingyi Wu, Bing Yang, Yanping Hu.

**Supervision:** Jingyi Wu, Bing Yang, Yanping Hu.

**Validation:** Bing Yang, Yanping Hu.

**Visualization:** Bing Yang, Yanping Hu.

**Writing – original draft:** Yanping Hu.

**Writing – review & editing:** Yanping Hu.
